# PAAR Proteins Are Versatile Clips That Enrich the Antimicrobial Weapon Arsenals of Prokaryotes

**DOI:** 10.1128/mSystems.00953-21

**Published:** 2021-12-07

**Authors:** Zheng Zhang, Ya Liu, Peng Zhang, Jianing Wang, Dandan Li, Yue-zhong Li

**Affiliations:** a State Key Laboratory of Microbial Technology, Institute of Microbial Technology, Shandong University, Qingdao, China; b Suzhou Research Institute, Shandong University, Suzhou, China; Ocean University of China

**Keywords:** PAAR protein, toxic effector, contractile injection system, CIS, prokaryotic genomes, Earth Microbiome Project, EMP

## Abstract

Protein toxins secreted by prokaryotes have been found to affect the pathogenicity of pathogens or directly mediate antagonistic interactions between prokaryotes. PAAR proteins are important carriers of toxic effectors and are located at the forefront of either the type VI secretion system (T6SS) or the extracellular contractile injection system (eCIS). This study systematically investigated PAAR homologues and related toxic effectors. We found that PAAR homologues were divided into 8 types and 16 subtypes and distributed in 23.1% of bacterial genomes and 7.8% of archaeal genomes. PAAR proteins of all types fold into a highly similar conical structure, even from relatively diverse underlying sequences. PAAR homologues associated with different secretion systems display a mixed phylogenetic relationship, indicating that PAAR proteins from such a subtype can be assembled on either a T6SS or an eCIS. More than 1,300 PAAR-related toxic effector genes were identified; one PAAR subtype can be associated with toxins of over 40 families, and toxins from one family can be associated with more than 10 PAAR subtypes. A large-scale comparison of Earth Microbiome Project data and prokaryotic genomes revealed that prokaryotes encoding *PAAR* genes are widely present in diverse environments worldwide, and taxa encoding multiple *PAAR* gene copies exhibit a wider distribution in environments than other taxa. Overall, our studies highlighted that PAAR proteins are versatile clips loaded with antimicrobial toxin bullets for secretion weapons (T6SS and eCIS), greatly enriching the weapon arsenal of prokaryotes, which, often together with VgrG, help prokaryotes fight for survival advantages in crowded environments.

**IMPORTANCE** Infectious diseases caused by microbial pathogens are severe threats to human health and economic development. To respond to these threats, it is necessary to understand how microorganisms survive in and adapt to complex environments. Microorganic toxins, which are widely distributed in nature, are the key weapons in life domain interactions. PAAR proteins are important carriers of prokaryotic toxic effectors. We reveal the versatility of PAAR proteins between secretory systems and the massive diversity of toxic effectors carried by PAAR proteins, which helps prokaryotes enrich their arsenal and expand their ability to attack their neighbors. A large number of PAAR homologues and related toxic effectors enhance the survival competitiveness of prokaryotic populations. In conclusion, our work provides an example for large-scale analysis of the global distribution and ecological functions of prokaryotic functional genes.

## INTRODUCTION

Bacterial contractile injection systems (CISs) are macromolecular machines that share homology with the bacteriophage contractile tail for the transfer of cytoplasmic proteins either out of the cell into the surrounding milieu or directly into the cytoplasm of eukaryotic and prokaryotic cells, mediating intercellular communication and antagonism (1–4). Bacterial type VI secretion systems (T6SSs), are a class of CISs, since they inject toxic effectors into adjacent target cells, and their structural components anchor to the inner membrane (5). Unlike the action mode of T6SSs, extracellular CISs (eCISs), such as *Photorhabdus* virulence cassette devices, resemble headless phages to release themselves into the surroundings to bind to and inject into target host cells (1). PAAR proteins, as important structural components of CISs, are located at the top of the central spike mounted on the entire secretory structure. PAAR proteins, which are orthologues of the protein gp5.4 of T4 phage, sharpen the tip of the spike complex and are responsible for the initial event of creating an opening in the target cell envelope while also acting as the site of effector recruitment (6–8). Considering the observed crucial function of PAAR proteins in CISs, it is necessary and urgent to analyze and describe the diversity of PAAR proteins in detail.

Few toxic effectors associated with PAAR proteins have been reported thus far. These toxins have been found to affect the pathogenicity of pathogens or directly mediate interactions among bacteria, and the ways in which they interact with PAAR proteins can be roughly divided into two types: one class of toxins is directly fused as the C-terminal extended domain of the PAAR protein, for example, Tse6 and Tse7 of Pseudomonas aeruginosa, Rhs-CT1 to CT10 of Escherichia coli, Tne2 of Pseudomonas protegens, and Rhs1 and Rhs2 of Serratia marcescens, all of which possess an N-terminal PAAR domain but a C-terminal extension containing various toxin domains (9–16); the other class of toxins form complexes with PAAR proteins (maybe through chaperone assistance), such as IglF of Francisella tularensis, TseT of P. aeruginosa, and TseTBg of Burkholderia gladioli (8, 17, 18).

Here, we comprehensively analyzed the sequenced prokaryotic genomes, with the identified *PAAR* genes as a starting point, and exploited novel toxins and immunity proteins through large-scale bioinformatics analysis, which revealed a tremendous diversity of toxic effectors probably carried by PAAR proteins. We further explored the distributions of the *PAAR* genes in prokaryotes and compared them with Earth Microbiome Project (EMP) data. Our results highlight that PAAR proteins and associated toxins promote the environmental adaptation of prokaryotic strains.

## RESULTS

### *PAAR* genes are widely found in the genomes of prokaryotes.

We searched for all known proteins containing the PAAR domain in the NCBI nonredundant reference sequence (RefSeq) database. PAAR domains are defined based on the PAAR-like superfamily (cl21497, containing DUF4150) and DUF4280 superfamily (cl16620) (6, 19, 20). Under the condition that the RPS-BLAST E value was ≤0.01, we identified a total of 47,625 proteins containing the PAAR domain, of which 99.35% were from bacteria, 0.36% were from viruses, 0.21% were from eukaryotes, and only 0.08% were from archaea.

NCBI sets at least one reference or representative genome for each sequenced species, and these genomes usually have high assembly quality. We analyzed the distribution of PAAR protein-encoding genes in 5,808 reference or representative prokaryotic genomes and identified a total of 3,022 *PAAR* genes (see Data Set S1 in the supplemental material). At least one *PAAR* gene is encoded in 23.1% of the bacterial genomes and 7.8% of the archaeal genomes. At the phylum level, 39.3% of 2,072 *Proteobacteria* genomes, 32.1% of 602 *Fibrobacteres*, *Chlorobi*, and *Bacteroidetes* (FCB) group genomes, 8.9% of 2605 *Terrabacteria* group genomes and 8.7% of 231 *Euryarchaeota* genomes encode *PAAR* genes (see Table S1). Among the major classes of prokaryotes (with at least 100 genomes), the proportions of *Alphaproteobacteria*, *Betaproteobacteria*, *Gammaproteobacteria*, and the delta/epsilon subdivision genomes of *Proteobacteria* encoding *PAAR* genes are 26.8, 46.7, 49.9, and 33.0%, respectively; the proportion for the *Bacteroidetes*/*Chlorobi* group of the FCB group is 32.0%; and the proportions for *Firmicutes* and *Actinobacteria* of the *Terrabacteria* group are 7.0 and 9.6%. However, there are no *PAAR* genes encoded in *Tenericutes* of the *Terrabacteria* group.

10.1128/mSystems.00953-21.6 TABLE S1 Statistical data of *PAAR* genes in the reference or representative prokaryotic genomes. Download Table S1, DOCX file, 0.02 MB. Copyright © 2021 Zhang et al. 2021 Zhang et al. https://creativecommons.org/licenses/by/4.0/ This content is distributed under the terms of the Creative Commons Attribution 4.0 International license.

We also counted instances of multiple copies of the *PAAR* gene in the reference or representative prokaryotic genomes. No instances of multiple copies of the *PAAR* gene were found in archaeal genomes, while 45.4% of the bacterial genomes encoding *PAAR* had multiple copies of the *PAAR* gene. The maximum copy number of the *PAAR* gene in a single genome was 35, which was observed in Chondromyces apiculatus DSM 436 of *Myxococcales* of *Proteobacteria*. Fifty percent of the *Proteobacteria* encoding the *PAAR* gene encode multiple copies, and the values are 53.9% for the FCB group and 25.3% for the *Terrabacteria* group. At the class level, 30.6, 67.4, 54.4, and 46.5% of the genomes of *Alphaproteobacteria*, *Betaproteobacteria*, *Gammaproteobacteria*, and delta/epsilon subdivisions of *Proteobacteria* encoding PAAR encode multiple copies, respectively; the values are 54.5% for the *Bacteroidetes*/*Chlorobi* group of the FCB group and 38.2 and 17.8% for *Firmicutes* and *Actinobacteria* of the *Terrabacteria* group, respectively. In short, the *PAAR* gene widely exists in prokaryotic genomes, and multiple copies are often present.

### PAAR homologues can be segregated into 8 types.

**FIG 1:**
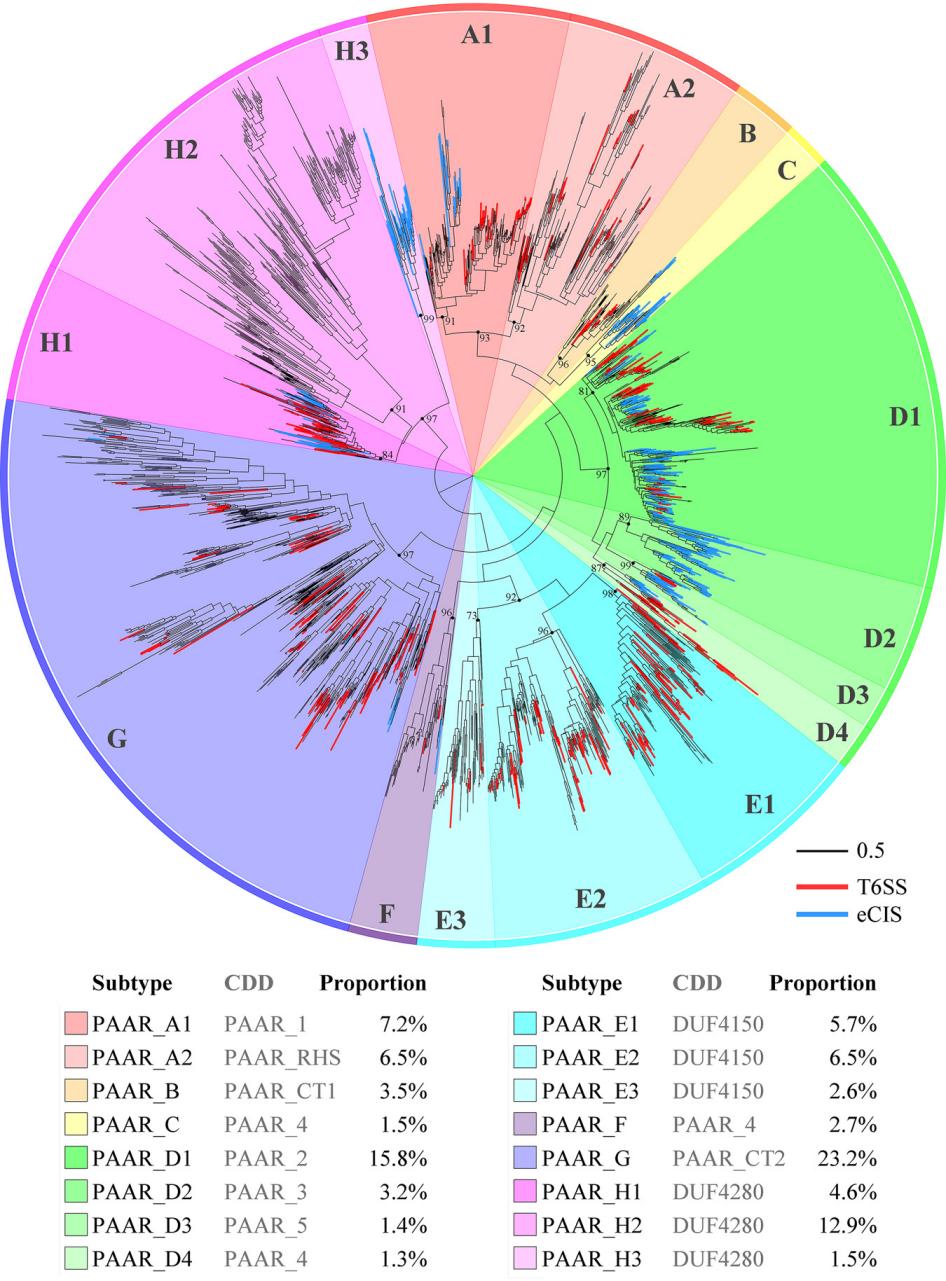
Phylogenetic relationship of PAAR homologues constructed based on the maximum-likelihood method. The phylogenetic relationship was constructed using only the sequences of the PAAR domain. PAAR homologues were divided into 8 types and 16 subtypes, which shared a certain correspondence with the CDD subfamily. Outer strips are color coded by the 8 PAAR types, and the inner background is colored according to the 16 subtypes. PAAR homologues related to T6SSs and eCISs are highlighted with red and blue branches, respectively.

We constructed a phylogenetic tree of the PAAR proteins based on the maximum-likelihood method by using the sequence of the PAAR domain, eliminating a few proteins with incomplete sequences (Fig. 1). The results showed that the PAAR proteins encoded by the reference or representative prokaryotic genomes can be classified into eight main phylogenetic clades, which are defined as eight PAAR types, and these eight types are further subdivided into 16 subtypes (PAAR_A1 to PAAR_H3). PAAR_A showed the closest phylogenetic relationship with PAAR_B, PAAR_C, and PAAR_D, followed by PAAR_E and PAAR_F and then by PAAR_G, whereas the PAAR_H (DUF4280) type was divergent from the other 7 types.

There is a certain correspondence between the PAAR subtypes and the PAAR subfamilies defined in the Conserved Domain Database (CDD), but the two are not completely consistent (Fig. 1). For example, the members of PAAR_A mostly have the PAAR_1 domain (cd14737, PAAR_A1) or PAAR_RHS domain (cd14742, PAAR_A2), while the members of PAAR_D include the PAAR_2 (cd14738, PAAR_D1), PAAR_3 (cd14739, PAAR_D2), PAAR_5 (cd14741, PAAR_D3) or PAAR_4 domain (cd14740, PAAR_D4). In particular, the PAAR_4 domain is present in three PAAR subtypes: PAAR_C, PAAR_D4, and PAAR_F. The members of PAAR_B, PAAR_E, PAAR_G, and PAAR_H basically correspond to PAAR_CT1 (cd14743), DUF4150 (pfam13665), PAAR_CT_2 (cd14744), and DUF4280 (pfam14107), respectively.

**FIG 2:**
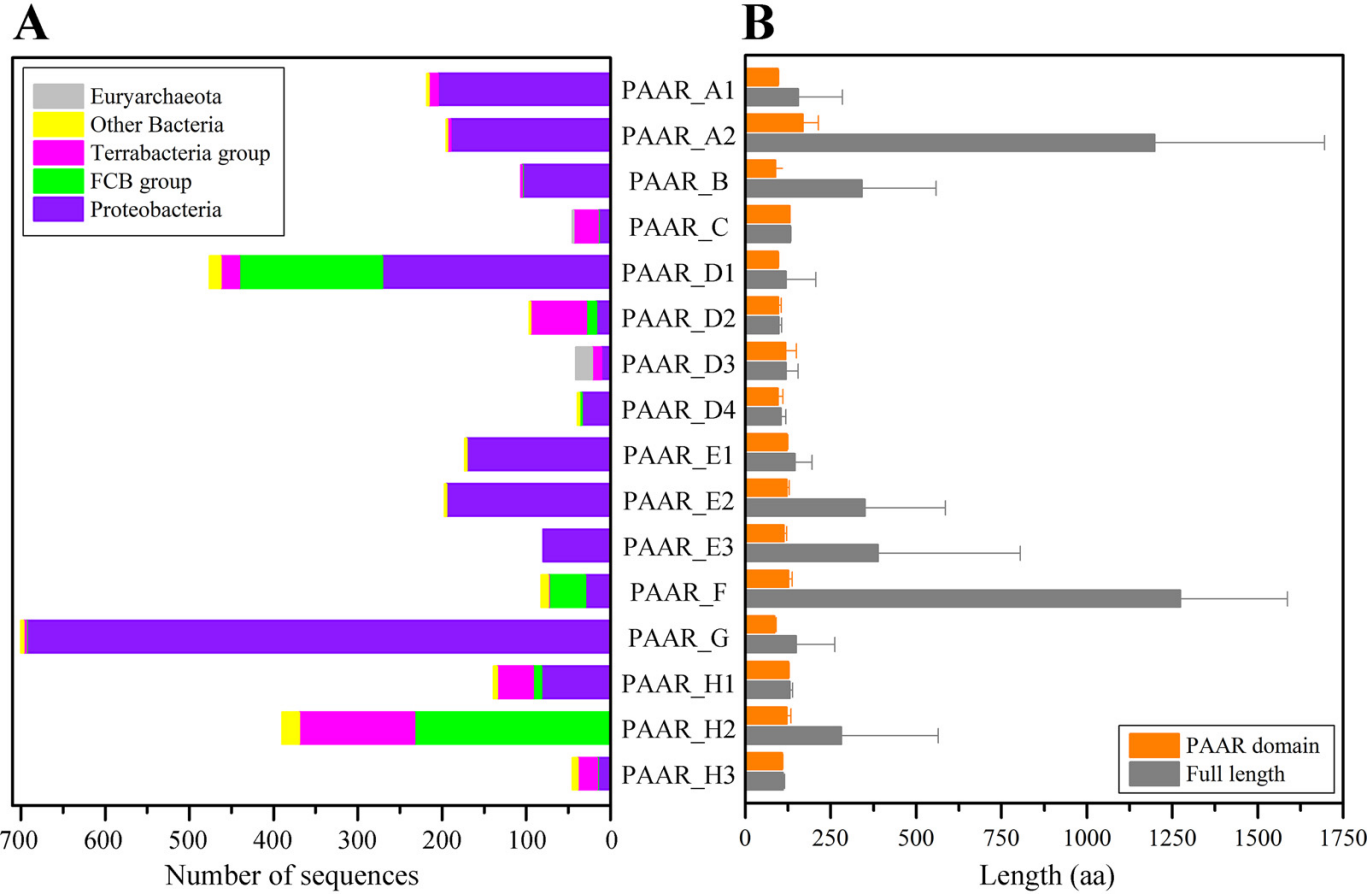
Species distributions and protein length comparison of the 16 PAAR subtypes. (A) Distributions of each PAAR subtype in prokaryotic genomes. (B) Comparison of the average length of each PAAR subtype, including the lengths of the PAAR domain and the whole protein.

In terms of quantity, the most abundant subtype PAAR_G accounts for 23.2% of all PAAR homologues, and the proportions of both PAAR_D1 and PAAR_H2 exceeds 10%, while the proportion of PAAR_C, PAAR_D3, PAAR_D4, or PAAR_H3 is only 1.3 to 1.5%. Regarding the source, Proteobacteria encode almost all the PAAR subtypes, except PAAR_H2, and the subtypes of PAAR_A, PAAR_B, PAAR_E, and PAAR_G are almost all encoded by *Proteobacteria* (Fig. 2A). The FCB group encode mainly PAAR_D, PAAR_F, and PAAR_H, the Gram-positive *Terrabacteria* group encode mainly PAAR_C, PAAR_D, and PAAR_H, and archaea encode only PAAR_D3. Regarding protein size, the average protein lengths of most of the PAAR subtypes do not exceed 150 amino acids (aa), which is close to the size of the PAAR domains. The results indicated that the members of these subtypes are mainly single PAAR domains (Fig. 2B). The average lengths of all proteins of the PAAR_B, PAAR_E2, PAAR_E3, and PAAR_H2 subtypes are between 250 and 400 aa, indicating that many of their members are multidomain proteins. The average protein lengths of PAAR_A2 and PAAR_F are more than 1,000 aa.

### Structural differences in PAAR subtypes.

**FIG 3:**
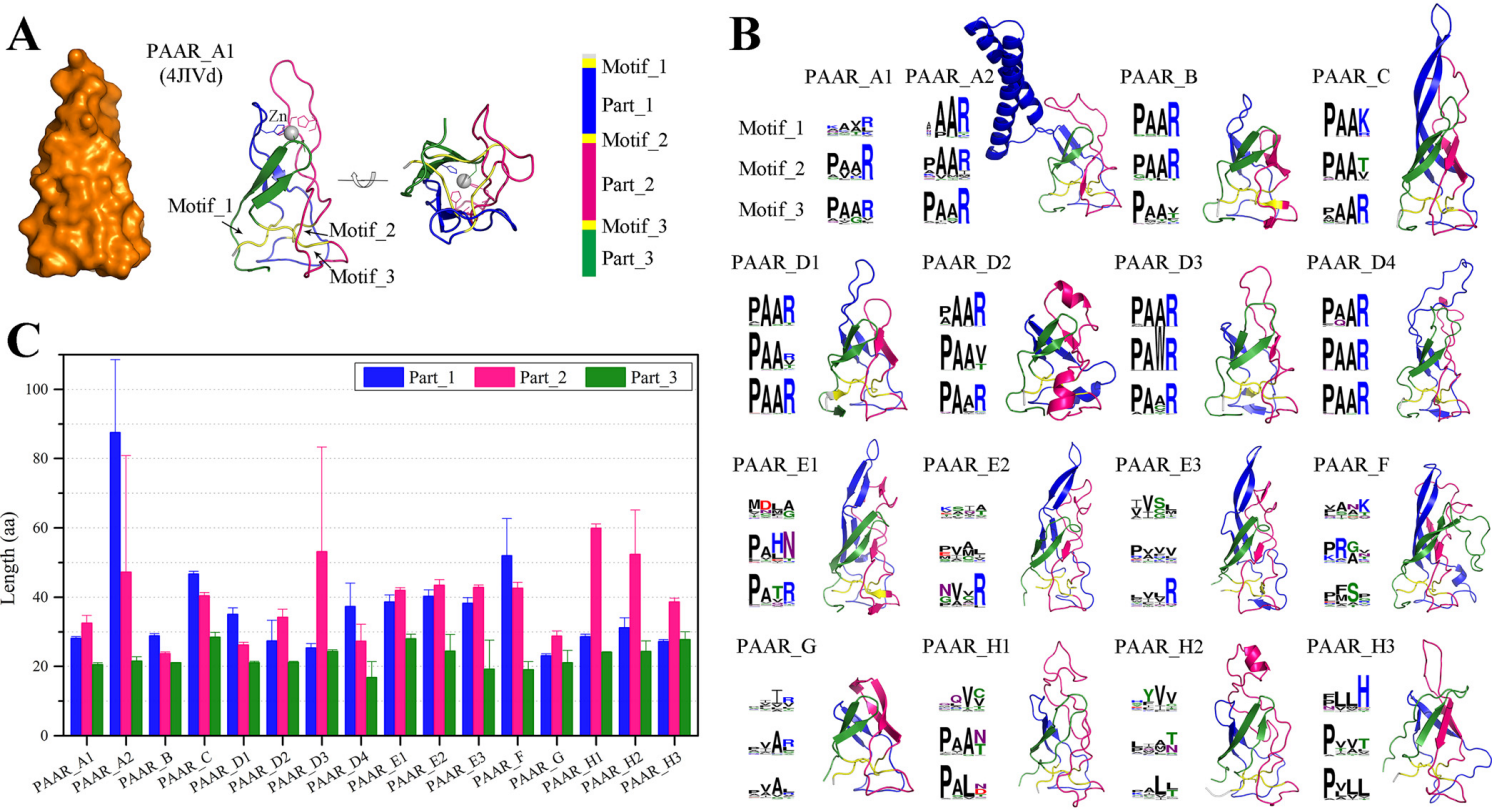
Structural characteristics of the 16 PAAR subtypes. (A) Crystal structure of the PAAR_A1 subtype (PDB 4JIVd) with three conserved PAAR motifs constituting the structural center. (B) Modeled structures and conserved motifs of different PAAR subtypes. (C) Comparison of the three parts of various PAAR subtypes.

Among the 16 PAAR subtypes, the structural characteristics of only the PAAR_A1 subtype were identified (Fig. 3A). The structures of representative proteins of the 15 other PAAR subtypes were modeled using the AlphaFold algorithm (see Table S2). This technology achieves accuracy competitive with that of experiments (21, 22). The results showed that the structural folding patterns of various PAAR subtypes were highly similar (Fig. 3B). Although the sequence identities between the modeled structures of the 15 PAAR subtypes and the crystal structures of the PAAR_A1 subtype are only 9 to 40%, the structural differences between them are small, with RMSD values not exceeding 3.3 Å and TM scores of not less than 0.6.

10.1128/mSystems.00953-21.7 TABLE S2 Structural modeling information of representative proteins of the 16 PAAR subtypes. Download Table S2, DOCX file, 0.02 MB. Copyright © 2021 Zhang et al. 2021 Zhang et al. https://creativecommons.org/licenses/by/4.0/ This content is distributed under the terms of the Creative Commons Attribution 4.0 International license.

The PAAR protein is conical, and three conserved PAAR motifs form the structural center for stable folding (Fig. 3A). Hydrophobic interactions and buried main chain hydrogen bonds mediate the interactions of the three PAAR motifs (6). The fold of the PAAR protein is stabilized by a Zn atom positioned close to the cone’s vertex, and the Zn-binding site consists of three histidines and one cysteine. Although the vertexes of these modeled structures are different due to the lack of metal atom binding, their structural centers are formed by the three regions of the polypeptide chain (PAAR motifs) (Fig. 3B). The sequences of the three PAAR motifs that constitute the center of the structure are most conserved in the four closely related types, namely, PAAR_A to PAAR_D, and a few sites can be replaced by amino acids with similar properties. In other types, typical conserved PAAR motifs are rarely observed, and only a few sites in the structural center were conserved.

Based on structural information and multiple sequence alignments, we determined the lengths of the various subtypes of PAAR domains (Fig. 2B). We found that the average length of the smallest PAAR subtype, PAAR_G, is 85.5 aa, while the average length of the largest PAAR subtype, PAAR_A2, is 167.9 aa. The three PAAR motifs that form the structural center (or the corresponding sites that form the structural center) are distant from each other in the sequence and separate the PAAR protein into three parts. The structural differences among PAAR subtypes manifest mainly as differences in the lengths of these three parts (Fig. 3C). For the smaller subtypes PAAR_B and PAAR_G, the lengths of Part_1, Part_2, and Part_3 are close to but not more than 30 aa. For the larger types PAAR_A2, PAAR_C, PAAR_E, and PAAR_F, the lengths of Part_1 and Part_2 increase simultaneously, while PAAR_D3 and PAAR_H have a large Part_2 of greater than 50 aa. Notably, the particularly long Part_1 (average length up to 87.5 aa) exists in PAAR_A2 and is been specialized to form hydrophobic helices in many proteins (see Fig. S1). The specialized hydrophobic helices contribute to translocating the toxin domain across the inner membrane to the target cell (15, 16).

10.1128/mSystems.00953-21.1 FIG S1 Modeled structures of whole proteins of Tse6 and Rhs1 belonging to the PAAR_A2 subtype. Inset shows the modeled structures of PAAR domains. Structures are shown as cartoons with color highlighting of N termini in blue and C termini in red. Download FIG S1, TIF file, 2.3 MB. Copyright © 2021 Zhang et al. 2021 Zhang et al. https://creativecommons.org/licenses/by/4.0/ This content is distributed under the terms of the Creative Commons Attribution 4.0 International license.

### Delivery modes of PAAR proteins.

**FIG 4:**
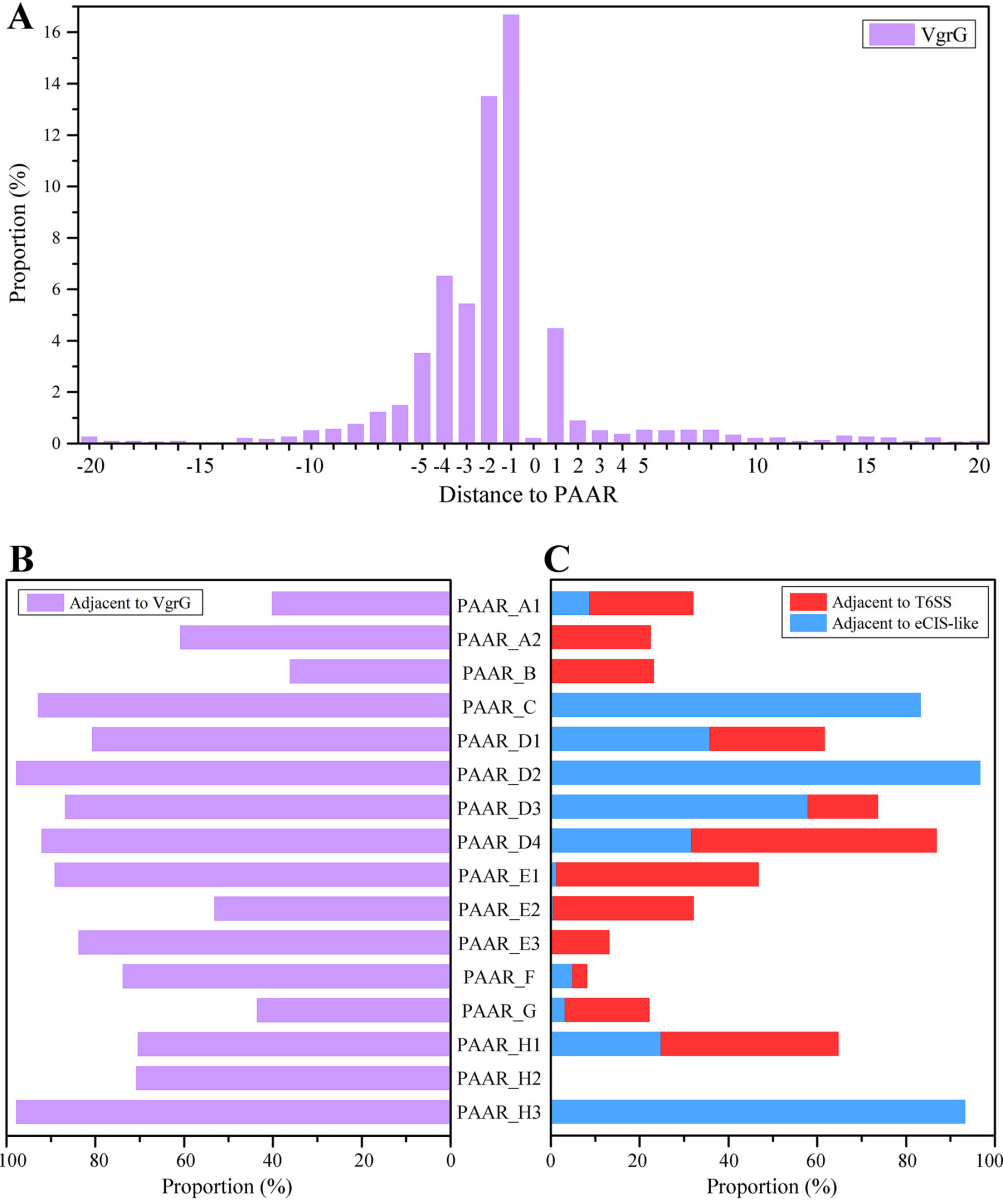
Potential delivery modes for PAAR proteins. (A) Proportions of 20 upstream or downstream genes of the *PAAR* genes encoding *vgrG* genes. The position of the *PAAR* gene was taken as 0, the position of the upstream gene of the *PAAR* gene is labeled with a negative value, and the position of the downstream gene is labeled with a positive value. (B) Proportions of each PAAR subtype adjacent to *vgrG* genes. (C) Proportions of each PAAR subtype adjacent to an eCIS (blue) and a T6SS (red).

PAAR proteins perform their functions by binding to VgrG proteins to form spike complexes (6). We found that the copy numbers of the *PAAR* genes encoded by the prokaryotic genomes are highly positively correlated with the copy numbers of the *vgrG* genes encoded by the prokaryotic genomes (Pearson’s *r *=* *0.81, *P < *0.01). For example, the strain Chondromyces apiculatus DSM 436, which encodes the highest number of *PAAR* gene copies in a single genome (35 copies), also encodes the highest number of *vgrG* gene copies (54 copies). In prokaryotic genomes, 45.6% of the *PAAR* genes have a *vgrG* gene within five upstream genes, 16.7% are directly adjacent to an upstream *vgrG* gene, and only 4.5% are adjacent to a downstream *vgrG* gene (Fig. 4A). With increasing distance from the *PAAR* gene, the occurrence rates of the *vgrG* genes decrease rapidly. In addition, 62.3% of the total *PAAR* genes have *vgrG* genes within 20 genes upstream or downstream, while the percentage is clearly different among the subtypes (Fig. 4B). For example, almost all genes encoding PAAR_D2 and PAAR_H3 are adjacent to *vgrG* genes, while only 36.2% of PAAR_B genes are adjacent to *vgrG* genes. It is worth noting that this proportion can differ considerably within the same type, such as 89.1% for PAAR_E1 and 53.2% for PAAR_E2.

Afp1/5 (Phage_T4_gp19), Afp2/3/4 (Phage_sheath_1), Afp11 (Baseplate_J), and Afp16 (DUF4255) are functionally critical to eCISs (3). The conserved components of T6SSs, namely, TssJ (T6SS-SciN), TssL (DotU), TssM (VI_IcmF), and ClpV (VI_ClpV1), do not exist in eCISs (23, 24). Therefore, we used the presence of genes encoding these conserved components upstream and downstream of a *PAAR* gene as the criterion for judging whether the *PAAR* gene was related to T6SSs or eCISs. Upon screening by the criteria, we found that at least 16.3% of the *PAAR* genes are associated with eCISs and 20.7% of the *PAAR* genes are associated with T6SSs, with no overlap between the two. Interestingly, some PAAR subtypes are associated with both eCISs and T6SSs (Fig. 4C). For example, the proportion of members related to eCISs or T6SSs exceeds 15% in each of the PAAR_D1, PAAR_D3, PAAR_D4, and PAAR_H1 subtypes. From the phylogenetic tree, we also noticed that many PAAR homologues that were related to eCISs or T6SSs had very close relationships (Fig. 1). For example, the two *PAAR* genes belonging to PAAR_D1 encoded by Desulfobacter curvatus DSM 3379 share an amino acid sequence identity as high as 63%, but one is related to an eCIS, while the other is related to a T6SS; the two *PAAR* genes belonging to PAAR_H1 encoded by Caballeronia glathei DSM 50014 exhibit 60% amino acid sequence identity and are also associated with an eCIS and a T6SS, respectively (see Fig. S2). These results indicated that one PAAR subtype can function with both eCISs and T6SSs. As components of eCISs or T6SSs, PAAR proteins exhibit no obvious differences in sequence and structural characteristics.

10.1128/mSystems.00953-21.2 FIG S2 Graphical depiction of gene neighborhoods for *PAAR* genes drawn to scale in four prokaryotic genomes. The bacterial species name and the PAAR subtype are indicated on the left. Gene neighborhoods are labeled with the corresponding information for conserved components of T6SSs or eCISs. Amino acid sequence identity of two PAAR proteins from the same species are indicated. Download FIG S2, TIF file, 0.5 MB. Copyright © 2021 Zhang et al. 2021 Zhang et al. https://creativecommons.org/licenses/by/4.0/ This content is distributed under the terms of the Creative Commons Attribution 4.0 International license.

In the CDD, VgrG homologues are classified into five superfamilies based on sequence differences, namely, Phage_GPD (cl15796), VgrG (cl34624), vgr_GE (cl36942), VI_Rhs_Vgr (cl37255), and VgrG_rel (cl41471). Because PAAR proteins and VgrG proteins are closely related in function, and their genes have a high cooccurrence within 20 genes upstream or downstream of the *PAAR* genes (Fig. 4B), we also analyzed the correspondence between PAAR subtypes and VgrG types (see Fig. S3). We found that there are 12 PAAR subtypes whose VgrG neighbors in the genome are mainly from single superfamilies (more than 70%). Furthermore, PAAR_D1 and PAAR_D4 are mainly related to VgrG homologues from two superfamilies, PAAR_G and PAAR_H2 are mainly related to VgrG homologues from three superfamilies. This result indicated that *PAAR* genes have a conserved evolutionary relationship with adjacent *vgrG* genes, but there are obviously some exceptions. Interestingly, in both PAAR_D1 and PAAR_D4, members related to T6SSs are all adjacent to VI_Rhs_Vgr (cl37255) in the genome, while members related to eCISs are all adjacent to vgr_GE (cl36942). In addition, homologues from Phage_GPD (cl15796) are mainly adjacent to PAAR_H2 members in the Gram-positive *Firmicutes* genomes, and conserved genes of eCISs or T6SSs are not present upstream and downstream. It is speculated that Gram-positive bacteria may possess an unknown phage-like translocation system.

10.1128/mSystems.00953-21.3 FIG S3 Superfamily classification of VgrG homologues that are associated with each PAAR subtype. The proportions of each PAAR subtype adjacent to *vgrG* genes are shown in Fig. 4B. *vgrG* genes were identified within 20 genes upstream or downstream of the *PAAR* genes. The classification of VgrG homologues is based on five CDD superfamilies. The abscissa is displayed as a percentage. Download FIG S3, TIF file, 0.7 MB. Copyright © 2021 Zhang et al. 2021 Zhang et al. https://creativecommons.org/licenses/by/4.0/ This content is distributed under the terms of the Creative Commons Attribution 4.0 International license.

In addition to the eCIS or T6SS component genes, the neighbors surrounding the *PAAR* genes also include some other associated genes. For example, the proportions of members related to eCISs in PAAR_C, PAAR_D2, and PAAR_H3 all exceed 80%, and the surrounding genes also have a high probability (more than 50%) of encoding DUF4157 in addition to encoding eCIS gene clusters. PAAR_A1, PAAR_B, PAAR_E, and PAAR_G are related mainly to T6SSs; the genes surrounding PAAR_E have a high probability of encoding DUF2169 (more than 75%), and some of the genes surrounding PAAR_A1 and PAAR_B encode DUF4123. Furthermore, members of PAAR_A2 and PAAR_F are rich in rearrangement hot spot (RHS) repeats. RHS proteins are a class of giant proteins representing a major group of secreted polymorphic toxins, and these proteins can be secreted through different routes, including T6SSs, to exert toxicity against target cells (9, 14, 25, 26). The genes surrounding more than 90% of PAAR_A2 members encode DcrB (DUF1795). T6SS adaptors/chaperones, including DUF4123-, DUF1795-, and potentially DUF2169-containing proteins, are required for loading specific effectors onto the cognate VgrG for delivering and stabilizing the effectors (27, 28). Finally, 70.8% of the PAAR_H2 genes are adjacent to *vgrG* genes, but we have not found that their surrounding genes encode eCIS or T6SS components. It is worth noting that some of the VgrG proteins related to PAAR_H2 contain the DUF2345 domain. DUF2345 is a conserved domain of uncharacterized proteins and is present in many VgrG proteins; this domain can be considered an extension of the spike region (29).

### PAAR proteins carry various toxins.

**FIG 5:**
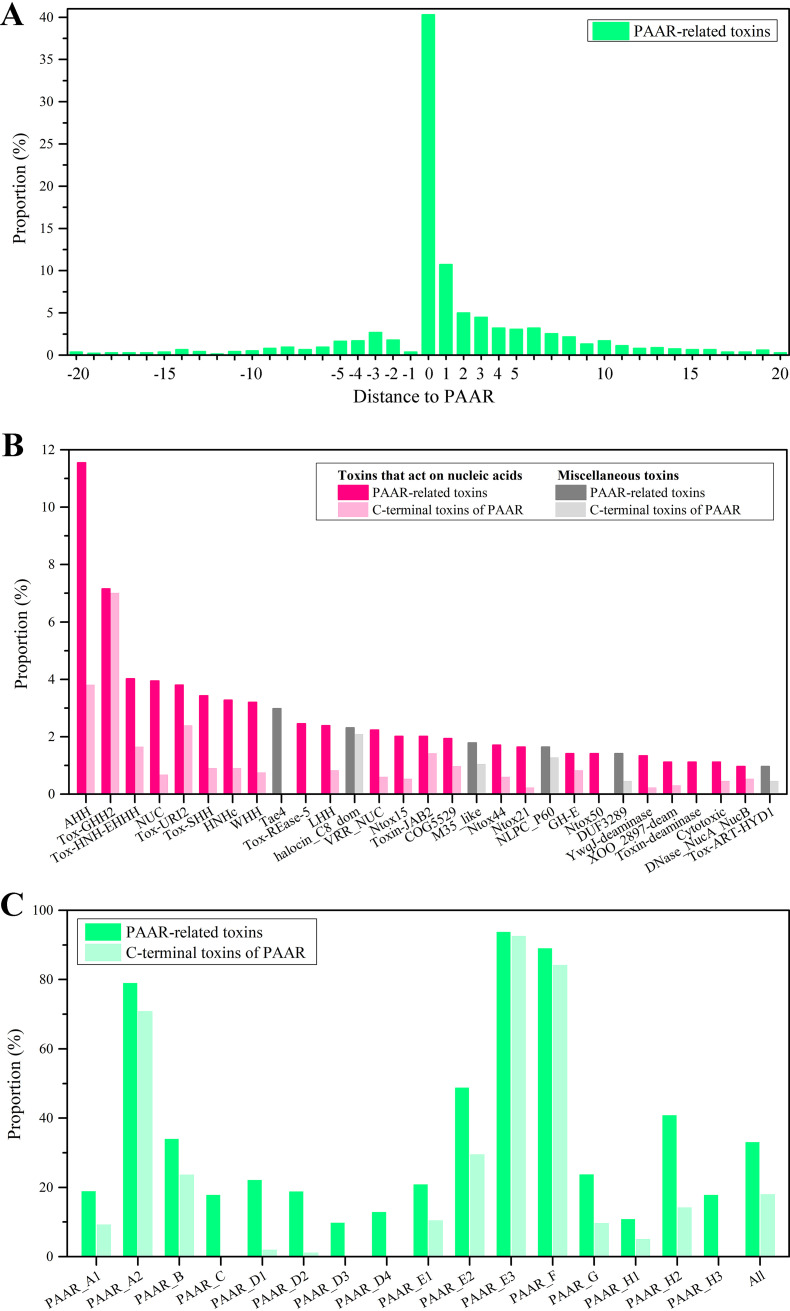
PAAR proteins carry a variety of toxins. (A) Proportions of genes within 20 genes upstream or downstream of the PAAR genes encoding toxins. The position of the *PAAR* gene was taken as 0, the position of the upstream gene of the *PAAR* gene is labeled with a negative value, and the position of the downstream gene is labeled with a positive value. (B) The PAAR-related toxins of 29 families that accounted for more than 1% of the total identified toxin proteins. Toxins whose activity is related to nucleases are colored pink, and other toxins are colored gray. All PAAR-related toxins are labeled with dark colors, and toxins located at the C terminus of the PAAR protein are labeled with light colors. (C) Proportion of members with PAAR-related toxin (dark green) or PAAR protein C-terminal toxin (light green) in each PAAR subtype.

PAAR proteins play an important role in toxic effector delivery by the CIS system, but only a few of the associated toxins have been identified. A large-scale scan of the *PAAR* genes and their surrounding genes encoding toxins in the reference or representative prokaryotic genomes identified >1,300 toxin genes (see Data Set S2). Of these toxins, 40.3% are encoded by *PAAR* genes as the C-terminal extension domain of the PAAR protein, and the rest are encoded by genes surrounding the PAAR genes (Fig. 5A). The toxin encoding probability of the genes of downstream PAAR is three times as high as that of upstream genes. Ten percent of the toxins are encoded by closely adjacent genes downstream of the *PAAR* gene, and as the distance from the *PAAR* genes increases, the probability of occurrence of toxin genes gradually decreases.

PAAR-related toxins from more than 80 protein families were identified, of which 59 families contained at least three toxin homologues (see Data Set S2). Most of these toxins are predicted to have enzymatic activity, and two-thirds of these enzymes are functionally related to nucleases. In particular, of the 29 toxin families that accounted for more than 1% of the total identified toxin proteins, 23 are functionally related to nucleases (Fig. 5B). The most common PAAR-related toxin is AHH nuclease, which accounts for 11.5% of the total toxins. We recently demonstrated its enzymatic activity and toxicity experimentally (20). In addition to nucleases, the activities of toxins include peptidase (such as M35_like), amidase (such as Tae4), and protein-modifying toxin (such as Tox-ART-HYD1) activities and a few unknown toxin activities (such as DUF3289). Most families of PAAR-related toxins can either be present as the C-terminal extension domains of PAAR proteins or are encoded by genes surrounding the *PAAR* gene. Only a few toxin families are nearly exclusively present as the C-terminal extension domains of PAAR proteins, such as Tox-GHH2 and halocin_C8_dom, or are encoded only by surrounding genes, such as Tae4 and Tox-REase-5.

At least 10% of the members of different PAAR subtypes have PAAR-related toxins, but there is a great difference among subtypes (Fig. 5C). At least 80% of the members of PAAR_A2, PAAR_E3, and PAAR_F have PAAR-related toxins, and almost all of these toxins are located at the C terminus of the PAAR proteins. Totally, at least one-third of the *PAAR* genes themselves or the surrounding genes encode toxins, and 7.4% of the *PAAR* genes are associated with at least two toxins. Each PAAR subtype includes various toxins (see Data Set S2); for example, the toxin proteins of 45 families are associated with PAAR_H2, and the toxins of more than 30 families are associated with PAAR_A2, PAAR_D1, and PAAR_G. Similarly, most types of toxins also correspond to multiple PAAR subtypes, such as HNHc, NUC, AHH, and Tox-HNH-EHHH, which are associated with more than 10 PAAR subtypes. Only a few toxins are related to a single PAAR subtype. For example, toxin-JAB2 corresponds mainly to PAAR_A2, Tox-REase-5 corresponds to PAAR_G, toxin-deaminase corresponds to PAAR_H2, and Tox-URI2 corresponds to PAAR_F.

### *PAAR* genes promote environmental adaptation of strains.

Our recent studies have shown that the genetic information of most prokaryotic biomes has been revealed to a high degree (30). The EMP samples the Earth’s microbial communities at an unprecedented scale to evaluate how prokaryotes are distributed in the global environment (31). We compared the genomic information of 5808 reference or representative prokaryotes with the sequence data of 10,000 EMP samples and determined the environmental distribution of these representative species based on 16S rRNA identity greater than 97%. As a result, 19,506 operational taxonomic units (OTUs) were identified. Although they accounted for only 7.4% of the total OTUs, the sequence abundance reached 50% of the total abundance.

We analyzed the environmental distribution of prokaryotes encoding *PAAR* genes based on the corresponding relationship between the reference or representative genomes and OTUs. Among 19,506 OTUs, 26.3% correspond to the genomes encoding *PAAR* genes (from 10 phyla and 20 classes), and 3.2% encode at least five copies (see Data Set S3). Furthermore, the EMP divides samples from different environments into 17 environmental labels (31). We found that the proportions of OTUs encoding *PAAR* genes in all the environmental types were not less than 20%, and the highest proportion was 33.0% in the plant rhizosphere (see Fig. S4A). Except for the hypersaline environment (for which only 13 samples were available), the proportions of OTUs encoding at least five copies of the *PAAR* genes in all the environmental types are 2.3 to 5.5%. These results indicated that prokaryotes encoding *PAAR* genes are widely distributed in various environments worldwide.

10.1128/mSystems.00953-21.4 FIG S4 Proportions of OTUs encoding *PAAR* genes (A) and *vgrG* genes (B) in 17 environment types. Download FIG S4, TIF file, 2.4 MB. Copyright © 2021 Zhang et al. 2021 Zhang et al. https://creativecommons.org/licenses/by/4.0/ This content is distributed under the terms of the Creative Commons Attribution 4.0 International license.

**FIG 6:**
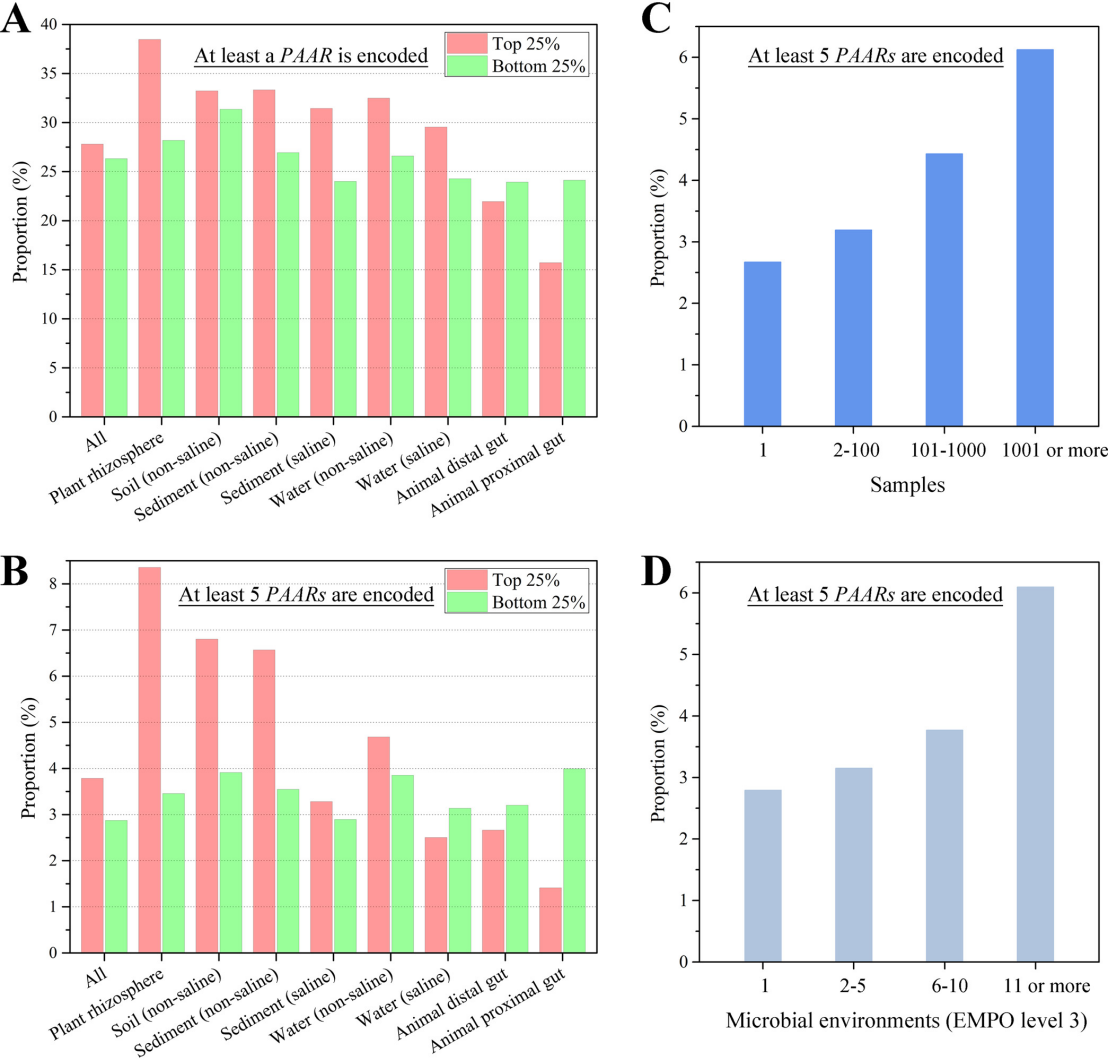
*PAAR* genes promote the environmental adaptation of bacterial strains. (A) Proportions of generalists and specialists that encode at least one *PAAR* gene. (B) Proportions of generalists and specialists that encode at least five *PAAR* genes. OTUs with the highest sequence abundance (top 25%) in each environment are called generalists (red), and OTUs with the lowest sequence abundance (bottom 25%) are called specialists (green). (C) Proportions of OTUs encoding at least five copies of the *PAAR* gene among OTUs that appeared in only a single sample, OTUs present in 2 to 100 samples, OTUs present in 101 to 1,000 samples, or OTUs present in more than 1,000 samples. (D) Proportions of OTUs encoding at least five copies of the *PAAR* gene among OTUs that survive in only a single environment type, OTUs that can survive in 2 to 5 types of environments, OTUs that can survive in 6 to 10 types of environments, and OTUs that can survive in more than 10 types of environments.

The difference in the number of genes encoding PAARs between OTUs with the highest sequence abundance (top 25%), i.e., generalists, and OTUs with the lowest sequence abundance (bottom 25%), i.e., specialists, in each environment was compared. The results showed that in the plant rhizosphere, with the highest proportion of OTUs encoding *PAAR* genes, 38.5% of generalists encode *PAAR* genes, while only 28.2% of specialists encode *PAAR* genes (Fig. 6A). The soil (nonsaline), sediment (nonsaline), sediment (saline), water (nonsaline), water (saline), and other free-living biomes also exhibit similar phenomena, indicating that the taxa encoding *PAAR* genes in these environments have relatively high abundance and are better adapted to the different environments. In contrast, in the animal proximal gut and animal distal gut, the proportion of generalists encoding *PAAR* genes is lower than that of specialists. In particular, in the animal proximal gut, only 15.7% of generalists encode *PAAR* genes, while 24.1% of specialists encode *PAAR* genes. The above phenomenon is more obvious for OTUs encoding at least 5 *PAAR* genes (Fig. 6B). In the plant rhizosphere, 8.4% of generalists encode at least 5 *PAAR* genes, while only 3.5% of specialists encode at least 5 *PAAR* genes, representing a difference of more than 2-fold. Therefore, encoding *PAAR* genes, especially encoding multiple copies of *PAAR* genes, allowed prokaryotic taxa in free-living biomes to strive for survival advantages.

The larger the sample number of OTU distributions is or the greater the number of types of environments in which an OTU can survive is, the stronger the ability of the OTU to adapt to different environments (32). We found that among the OTUs that appeared in only a single sample, the proportion of OTUs encoding at least five copies of *PAAR* genes is 2.7% (Fig. 6C). For OTUs present in 2 to 100 samples, this proportion rises to 3.2%; for OTUs present in 101 to 1,000 samples, the value is 4.4%; for OTUs present in more than 1,000 samples, this value reaches 6.1%. Similarly, for OTUs that survived in only a single environmental type, the proportion of OTUs that encoded at least five copies of the *PAAR* genes is 2.8% (Fig. 6D). For OTUs that can survive in two to five types of environments, this proportion rises to 3.2%; for OTUs that can survive in 6 to 10 types of environments, this value is 3.8%; for OTUs that can survive in more than 10 types of environments, this value reaches 6.1%. Remarkably, the most widespread OTUs encoding PAARs (present in more than 10 environment types) are not concentrated in a few taxa but come from as many as 6 phyla and 12 classes. Therefore, widespread prokaryotic taxa encode more *PAAR* genes in their genomes than less widely distributed taxa.

Furthermore, we also focused on the environmental distribution of prokaryotes encoding *vgrG* genes (see Fig. S4B). The results showed that the proportions of OTUs encoding *vgrG* genes are not less than 25% in all the environmental types and are highly positively correlated with the proportions of OTUs encoding *PAAR* genes (Pearson’s *r *=* *0.99, *P* < 0.01). Similar to the results for *PAAR* genes, the proportions of generalists encoding *vgrG* genes are higher than those of specialists in free-living prokaryotic biomes, while the opposite is true in the animal gut environment (see Fig. S5A and B). In addition, widespread prokaryotic taxa also encode more *vgrG* genes in their genomes than less widely distributed taxa (see Fig. S5C and D). In summary, the results indicated that the protein translocation systems composed of PAAR, VgrG, etc., are closely related to the survival competitiveness of strains.

10.1128/mSystems.00953-21.5 FIG S5 *vgrG* genes are related to the environmental adaptation of bacterial strains. Proportions of generalists and specialists that encode at least one *vgrG* gene (A) and at least five *vgrG* genes (B). Proportions of OTUs encoding at least five copies of the *vgrG* gene among OTUs that appeared in only a single sample, 2 to 100 samples, 101 to 1,000 samples, and more than 1,000 samples (C) or among OTUs that survive in only a single environment type, 2 to 5 types of environments, 6 to 10 types of environments, and more than 10 types of environments (D). Download FIG S5, TIF file, 1.2 MB. Copyright © 2021 Zhang et al. 2021 Zhang et al. https://creativecommons.org/licenses/by/4.0/ This content is distributed under the terms of the Creative Commons Attribution 4.0 International license.

## DISCUSSION

PAAR proteins are important carriers of toxic effectors located at the front of the prokaryotic contractile injection system complex (6). However, compared to the attention given to other toxic effector carriers, such as VgrG, that given to the PAAR protein remains insufficient. Here, the role and importance of PAAR proteins in the context of general genomics were investigated with bioinformatics methods for the first time. More than 40,000 PAAR homologues were identified, of which no more than one-thousandth had been reported previously. The PAAR homologues can be divided into eight different types and subdivided into 16 subtypes. Their coding genes are widely distributed in prokaryotic genomes, and the copy numbers astonishingly reach as high as 35. The marked genetic diversity and wide distribution suggest that PAAR homologues play an important role in prokaryotes for their ecological functions.

We revealed the prevalence of PAARs in prokaryotes and their close ties with the bacterial injection machinery, including the T6SS and eCIS, which are systems that major contributors to bacterial competition. According to observations made by cryo-electron tomography, PAAR proteins can be used as a spike protein of T6SSs or eCISs (1, 4). Interestingly, we found that there is no clear boundary between the PAAR proteins associated with T6SSs and eCISs in terms of phylogenetic relationship. Some PAAR subtypes can function with both T6SSs and eCISs. The versatility of PAAR proteins in secretory systems expands the ability of bacteria to attack surrounding neighbors. We also observed a great diversity of toxins associated with PAAR proteins. One PAAR subtype can be associated with more than 40 types of toxins, and one type of toxin can be associated with more than 10 PAAR subtypes. If a T6SS or an eCIS is regarded as a gun loaded with antibacterial toxin bullets, PAAR proteins are similar to versatile clips. On the one hand, the clips can be used for two secretion system weapons; on the other hand, the clips can carry a variety of toxin bullets. Therefore, PAAR proteins greatly enrich the arsenal of prokaryotes.

Because microorganic toxins exhibit high diversity in sequence and function, identifying unknown toxins is challenging. Our recent work demonstrated an effective approach of using adaptors as markers to identify toxic effectors (33). Furthermore, some toxic effectors associated with PAAR proteins were also reported in our previous work (13, 20). In this study, we performed a comprehensive analysis of sequenced prokaryotic genomes, starting with the identification of *PAAR* genes, and expanded novel toxins and immunity proteins with large-scale bioinformatics methods. In addition, 1,343 potential toxin genes from 84 different families were identified, and two-thirds of these are currently annotated as hypothetical proteins or as having unknown functions. Our results revealed the diversity of toxic effectors carried by PAAR proteins and further increased the understanding of prokaryotic antimicrobial toxin systems.

The genetic information of most prokaryotic biomes has been revealed to a high degree (30). Based on EMP data, we determined that microorganisms encoding *PAAR* genes are widely present in various environments worldwide. Encoding PAAR genes, especially multiple copies of *PAAR* genes, helps prokaryotic taxa in free-living biomes acquire survival advantages but is not conducive to survival in host-associated biomes. We speculated that the possible reasons for this phenomenon were as follows: on the one hand, the alpha diversity of prokaryotic taxa in the free-living biomes was higher, and the competition among taxa was more intense, so the roles of PAAR proteins and related toxins were very important; on the other hand, many PAAR-related toxins could also act on eukaryotic cells, and a large number of *PAAR* genes and related toxins were encoded to cause harm to the host. Similarly, we found that the environmental distribution of VgrG homologues was highly positively correlated with that of PAAR homologues. The presence of a large number of *PAAR* genes, *vgrG* genes and involved toxin genes is closely related to enhancing the survival competitiveness of the population.

In general, the findings will be of great interest to the protein secretion system field and also those interested in microbial interactions more broadly. This research may help clarify the interaction mechanism of microorganisms in the community, and the results may also have reference value in disease prevention and treatment. Our work provides an example of a large-scale analysis of the global distribution and ecological functions of prokaryotic functional genes.

## MATERIALS AND METHODS

### Acquisition of PAAR homologues.

Taxonomic information and functional annotation information of the protein domain were acquired from the CDD and PFAM database (34, 35). PAAR domains are defined based on the PAAR-like superfamily (cl21497, containing DUF4150) and DUF4280 superfamily (cl16620) (6, 19, 20). All proteins with PAAR domains were identified in the CDD (52,910 position-specific scoring matrices [PSSMs]) by RPS-BLAST, and the retrieval condition was that the E value did not exceed 0.01. Taxonomic information of species was obtained from the NCBI taxonomic database.

The NCBI defines at least one reference or representative genome for each sequenced species. These genomes usually have high sequencing quality. The genome sequence information of all the reference or representative prokaryotes and the positional information of all the coding genes in the genome were obtained from the NCBI RefSeq database (36). The identified PAAR proteins were correlated with the genomic protein products through their accession number to determine the *PAAR* genes in each genome.

### Phylogenetic analysis of PAAR domains.

The sequences of PAAR domains were extracted from the CDD and employed to construct the initial phylogenetic relationship by domain annotation. Multiple alignment of amino acid sequences of all PAAR domains was implemented using MAFFT (FFT-NS-i, BLOSUM62) (37). A maximum-likelihood tree was constructed using FastTree with the JTT+CAT model (38). The reliability of the corresponding split in the tree was calculated with the Shimodaira-Hasegawa test (39). The phylogenetic tree was visualized by iTOL (40).

Based on the initial phylogenetic relationship, representative sequences were selected in the main clusters for structural modeling. Based on the structural information and multiple sequence alignments of each type of PAAR protein, the start and end positions of the PAAR domains were modified, and a few proteins with incomplete domain sequences were eliminated. Based on all the modified PAAR domain sequences, multiple sequence alignments were performed again, and phylogenetic relationships were constructed again for final analysis.

### Modeling and display of PAAR structures.

AlphaFold is an AI system that predicts a protein’s three-dimensional structure from its amino acid sequence (21, 22). It regularly achieves accuracy competitive with that of experiments. The three-dimensional structures of PAAR proteins were modeled by AlphaFold and displayed by PyMOL (Schrödinger, LLC).

Structural differences in each of the modeled structures with the crystal structure were measured with TM-align (41). The TM-score, ranging from 0 to 1, was used to measure the structural similarity of two protein structures (42). The higher the TM-score was, the more similar the two aligned structures. If the TM-score was higher than 0.5, the pairwise structures were assumed to have the same fold (43).

Multiple structure alignments of the crystal and all the modeled structures of PAAR proteins were implemented in VMD software (44). The positions of the three PAAR motifs in each modeling structure were determined based on the corresponding positions in the crystal structure. According to the positions of the three motifs in each PAAR subtype, the sequences were divided into three parts: Part_1 was located between Motif_1 and Motif_2, Part_2 was located between Motif_2 and Motif_3, and Part_3 was located from Motif_3 to the C terminus. The conservation of PAAR motifs in each subtype is illustrated by multiple sequence alignment and the sequence logo (45).

### Genomic context analysis.

Context analysis was performed for each of the reference or representative prokaryotic genomes to extract 20 adjacent genes upstream or downstream of each *PAAR* gene. CDD domain annotations of the protein products of all genes were performed with an E value threshold of 0.01 (35).

The eCIS components Afp11, Afp12, Afp13, Afp14, and Afp16 are not seen in T6SSs, but Afp11 and Afp16 are encoded in more than 85% of eCIS gene clusters (3). Single-gene knockout experiments indicated that Afp2/3/4, Afp1/5, and Afp11 are essential for eCIS function (46). Homologues of Afp1/5 (Phage_T4_gp19), Afp2/3/4 (Phage_sheath_1), Afp11 (Baseplate_J), and Afp16 (DUF4255) from the corresponding CDD superfamilies were not found in T6SSs. Therefore, a *PAAR* gene was determined to be related to eCISs based on the presence of at least two of the four homologues encoding Afp1/5, Afp2/3/4, Afp11, and Afp16 among the 20 genes upstream or downstream of the *PAAR* gene. Similarly, the conserved components TssJ, TssL, and TssM (transenvelope complex) and ClpV (sheath recycling) of the T6SS are not present in eCISs (4, 23, 24). Therefore, a *PAAR* gene was determined to be related to T6SSs if at least two of the four homologues encoding TssJ (T6SS-SciN), TssL (DotU), TssM (VI_IcmF), and ClpV (VI_ClpV1) were present among the 20 genes upstream and downstream of the *PAAR* gene.

The correspondence between PAAR subtypes and VgrG types was determined by identifying the presence of *vgrG* genes within 20 genes upstream or downstream of the *PAAR* genes. The classification of VgrG homologues is based on five superfamilies: Phage_GPD (cl15796), VgrG (cl34624), vgr_GE (cl36942), VI_Rhs_Vgr (cl37255), and VgrG_rel (cl41471).

In the CDD, the collected toxic effector proteins were divided into 112 families, and the immunity proteins were divided into 92 families. We identified homologues of these toxin families and immunity protein families in the protein products of all the *PAAR* genes and the 20 upstream or downstream genes. Since many homologues of the immunity protein families were not well identified, we supplemented them by BLASTp alignment using the identified immunity proteins as seeds. The threshold of the E value for the BLASTp alignment was less than 0.001. If a protein had some other functions in addition to the toxic effector function, the protein was recognized as a toxin only if its homologue was located at the C terminus of the PAAR protein or its coding gene was close to downstream of the immunity protein-coding gene.

### Environmental distribution of *PAAR* genes.

The EMP was founded in 2010 to sample the Earth’s microbial communities to advance our understanding of the organizing biogeographic principles that govern microbial community structure on Earth (31). A total of 262,011 OTUs were obtained from a set of 10,000 EMP samples using Deblur software (47). Chimaera filtering relied on the EMP project.

Alignment between the EMP OTUs and 5808 reference or representative prokaryotic genomes was performed using BLASTn, and the corresponding relationship was determined with 16S rRNA (V4 region) identity greater than 97% as the standard. We analyzed the environmental distribution of prokaryotes encoding *PAAR* genes or *vgrG* genes based on the corresponding relationship between the reference or representative genomes and OTUs. The Earth Microbiome Project Ontology (EMPO) classified 17 microbial environments (level 3) as free-living or host-associated (level 1) and saline or nonsaline (if free-living) or animal or plant (if host-associated) (level 2) (31).

10.1128/mSystems.00953-21.8 DATA SET S1 Distribution of *PAAR* genes in the reference or representative prokaryotic genomes. Download Data Set S1, XLSX file, 0.1 MB. Copyright © 2021 Zhang et al. 2021 Zhang et al. https://creativecommons.org/licenses/by/4.0/ This content is distributed under the terms of the Creative Commons Attribution 4.0 International license.

10.1128/mSystems.00953-21.9 DATA SET S2 Information on PAAR-related toxins in the reference or representative prokaryotic genomes. Download Data Set S2, XLSX file, 0.1 MB. Copyright © 2021 Zhang et al. 2021 Zhang et al. https://creativecommons.org/licenses/by/4.0/ This content is distributed under the terms of the Creative Commons Attribution 4.0 International license.

10.1128/mSystems.00953-21.10 DATA SET S3 Summary of OTUs encoding *PAAR* genes in EMP data. Download Data Set S3, XLSX file, 0.6 MB. Copyright © 2021 Zhang et al. 2021 Zhang et al. https://creativecommons.org/licenses/by/4.0/ This content is distributed under the terms of the Creative Commons Attribution 4.0 International license.
