# Supplementary material for: PAAR Proteins Are Versatile Clips That Enrich the Antimicrobial Weapon Arsenals of Prokaryotes
Source: mSystems. 2021 Dec 7;6(6):e00953-21. doi: 10.1128/mSystems.00953-21 (PMC8651086; doi:10.1128/mSystems.00953-21)
Supplement: TABLE S1 [file msystems.00953-21-st001.docx]

**Supplementary table 1.** Statistical data of *PAAR* genes in the reference or representative prokaryotic genomes.

| **Taxa** | | **All genomes** | **Genomes with *PAAR*genes (proportion)** | **Genomes with multiple copies**  **of *PAAR* (proportion)** |
| --- | --- | --- | --- | --- |
| **Phyla** | | | | |
| Archaea | Euryarchaeota | 231 | 20 (8.7%) | 0 |
| Archaea | TACK group | 26 | 0 | 0 |
| Bacteria | Acidobacteria | 18 | 7 (38.9%) | 4 (57.1%) |
| Bacteria | Aquificae | 14 | 1 (7.1%) | 0 |
| Bacteria | Caldiserica/Cryosericota group | 1 | 0 | 0 |
| Bacteria | Calditrichaeota | 1 | 0 | 0 |
| Bacteria | Chrysiogenetes | 2 | 0 | 0 |
| Bacteria | Coprothermobacterota | 2 | 0 | 0 |
| Bacteria | Deferribacteres | 5 | 0 | 0 |
| Bacteria | Dictyoglomi | 2 | 0 | 0 |
| Bacteria | Elusimicrobia | 3 | 0 | 0 |
| Bacteria | FCB group | 602 | 193 (32.1%) | 104 (53.9%) |
| Bacteria | Fusobacteria | 26 | 14 (53.8%) | 3 (21.4%) |
| Bacteria | Nitrospinae/Tectomicrobia group | 1 | 0 | 0 |
| Bacteria | Nitrospirae | 10 | 2 (20.0%) | 1 (50.0%) |
| Bacteria | Proteobacteria | 2072 | 814 (39.3%) | 407 (50.0%) |
| Bacteria | PVC group | 58 | 12 (20.7%) | 3 (25.0%) |
| Bacteria | Spirochaetes | 69 | 4 (5.8%) | 1 (25.0%) |
| Bacteria | Synergistetes | 16 | 2 (12.5%) | 0 |
| Bacteria | Terrabacteria group | 2605 | 233 (8.9%) | 59 (25.3%) |
| Bacteria | Thermodesulfobacteria | 9 | 0 | 0 |
| Bacteria | Thermotogae | 26 | 0 | 0 |
| Bacteria | unclassified Bacteria | 9 | 1 (11.1%) | 0 |
| **Class** | | | | |
| Acidobacteria | Acidobacteriia | 13 | 6 (46.2%) | 4 (66.7%) |
| Acidobacteria | Blastocatellia | 2 | 0 | 0 |
| Acidobacteria | Holophagae | 2 | 1 (50.0%) | 0 |
| Acidobacteria | Thermoanaerobaculia | 1 | 0 | 0 |
| Aquificae | Aquificae | 14 | 1 (7.1%) | 0 |
| Caldiserica/Cryosericota group | Caldiserica | 1 | 0 | 0 |
| Calditrichaeota | Calditrichae | 1 | 0 | 0 |
| Chrysiogenetes | Chrysiogenetes | 2 | 0 | 0 |
| Coprothermobacterota | Coprothermobacteria | 2 | 0 | 0 |
| Deferribacteres | Deferribacteres | 5 | 0 | 0 |
| Dictyoglomi | Dictyoglomia | 2 | 0 | 0 |
| Elusimicrobia | Elusimicrobia | 1 | 0 | 0 |
| Elusimicrobia | Endomicrobia | 1 | 0 | 0 |
| Elusimicrobia | environmental samples | 1 | 0 | 0 |
| Euryarchaeota | Stenosarchaea group | 148 | 20 (13.5%) | 0 |
| Euryarchaeota | Methanomada group | 38 | 0 | 0 |
| Euryarchaeota | Thermococci | 27 | 0 | 0 |
| Euryarchaeota | Diaforarchaea group | 10 | 0 | 0 |
| Euryarchaeota | Archaeoglobi | 7 | 0 | 0 |
| Euryarchaeota | Methanonatronarchaeia | 1 | 0 | 0 |
| FCB group | Bacteroidetes/Chlorobi group | 597 | 191 (32.0%) | 104 (54.5%) |
| FCB group | Fibrobacteres | 3 | 0 | 0 |
| FCB group | Gemmatimonadetes | 2 | 2 (100.0%) | 0 |
| Fusobacteria | Fusobacteriia | 26 | 14 (53.8%) | 3 (21.4%) |
| Nitrospinae/Tectomicrobia group | Nitrospinae | 1 | 0 | 0 |
| Nitrospirae | Nitrospira | 10 | 2 (20.0%) | 1 (50.0%) |
| Proteobacteria | Gammaproteobacteria | 835 | 417 (49.9%) | 227 (54.4%) |
| Proteobacteria | Alphaproteobacteria | 731 | 196 (26.8%) | 60 (30.6%) |
| Proteobacteria | Betaproteobacteria | 276 | 129 (46.7%) | 87 (67.4%) |
| Proteobacteria | delta/epsilon subdivisions | 215 | 71 (33.0%) | 33 (46.5%) |
| Proteobacteria | Oligoflexia | 6 | 0 | 0 |
| Proteobacteria | Acidithiobacillia | 5 | 1 (20.0%) | 0 |
| Proteobacteria | Hydrogenophilalia | 2 | 0 | 0 |
| Proteobacteria | Zetaproteobacteria | 2 | 0 | 0 |
| PVC group | Planctomycetes | 22 | 10 (45.5%) | 3 (30.0%) |
| PVC group | Chlamydiae | 17 | 0 | 0 |
| PVC group | Verrucomicrobia | 17 | 2 (11.8%) | 0 |
| PVC group | Kiritimatiellaeota | 1 | 0 | 0 |
| PVC group | Lentisphaerae | 1 | 0 | 0 |
| Spirochaetes | Spirochaetia | 69 | 4 (5.8%) | 1 (25.0%) |
| Synergistetes | Synergistia | 16 | 2 (12.5%) | 0 |
| TACK group | Crenarchaeota | 24 | 0 | 0 |
| TACK group | Thaumarchaeota | 2 | 0 | 0 |
| Terrabacteria group | Firmicutes | 1278 | 89 (7.0%) | 34 (38.2%) |
| Terrabacteria group | Actinobacteria | 1050 | 101 (9.6%) | 18 (17.8%) |
| Terrabacteria group | Tenericutes | 116 | 0 | 0 |
| Terrabacteria group | Cyanobacteria/Melainabacteria group | 84 | 23 (27.4%) | 6 (26.1%) |
| Terrabacteria group | Deinococcus-Thermus | 43 | 9 (20.9%) | 1 (11.1%) |
| Terrabacteria group | Chloroflexi | 31 | 11 (35.5%) | 0 |
| Terrabacteria group | Armatimonadetes | 2 | 0 | 0 |
| Terrabacteria group | unclassified Terrabacteria group | 1 | 0 | 0 |
| Thermodesulfobacteria | Thermodesulfobacteria | 9 | 0 | 0 |
| Thermotogae | Thermotogae | 26 | 0 | 0 |
| unclassified Bacteria | unclassified Bacteria (miscellaneous) | 8 | 1 (12.5%) | 0 |
| unclassified Bacteria | Haloplasmatales | 1 | 0 | 0 |
