# Supplementary material for: PAAR Proteins Are Versatile Clips That Enrich the Antimicrobial Weapon Arsenals of Prokaryotes
Source: mSystems. 2021 Dec 7;6(6):e00953-21. doi: 10.1128/mSystems.00953-21 (PMC8651086; doi:10.1128/mSystems.00953-21)
Supplement: TABLE S2 [file msystems.00953-21-st002.docx]

**Supplementary table 2.** Structural modelling information of representative proteins of the 16 PAAR subtypes.

| **Refseq accession** | **PAAR subtype** | **Length (aa)** | | | | **Structure comparisons with 4JIVd** | | | | |  |
| --- | --- | --- | --- | --- | --- | --- | --- | --- | --- | --- | --- |
|  |  | **Whole protein** | **PAAR domain** | **Start** | **End** | | **TM-score** | **RMSD** | **IDEN** | **Cov** | |
| WP_000526403.1  (4JIVd) | PAAR_A1 | 94 | 94 | 1 | 94 | |  |  |  |  | |
| WP_003115074.1 | PAAR_A2 | 430 | 158 | 4 | 161 | | 0.83 | 2.31 | 0.30 | 0.97 | |
| WP_003104235.1 | PAAR_B | 131 | 88 | 3 | 90 | | 0.83 | 1.24 | 0.32 | 0.89 | |
| WP_044967090.1 | PAAR_C | 138 | 129 | 9 | 137 | | 0.89 | 1.50 | 0.29 | 0.99 | |
| WP_005499010.1 | PAAR_D1 | 96 | 94 | 2 | 95 | | 0.87 | 1.25 | 0.40 | 0.93 | |
| WP_012378964.1 | PAAR_D2 | 105 | 102 | 1 | 102 | | 0.79 | 1.88 | 0.28 | 0.91 | |
| WP_010941651.1 | PAAR_D3 | 98 | 98 | 1 | 98 | | 0.82 | 1.68 | 0.38 | 0.94 | |
| WP_043879337.1 | PAAR_D4 | 127 | 126 | 1 | 126 | | 0.88 | 1.71 | 0.31 | 0.99 | |
| WP_004934933.1 | PAAR_E1 | 130 | 128 | 1 | 128 | | 0.83 | 1.81 | 0.15 | 0.99 | |
| WP_011550193.1 | PAAR_E2 | 154 | 127 | 11 | 137 | | 0.77 | 2.15 | 0.19 | 0.96 | |
| WP_012241028.1 | PAAR_E3 | 469 | 126 | 5 | 130 | | 0.79 | 2.10 | 0.19 | 0.95 | |
| WP_012444473.1 | PAAR_F | 1579 | 150 | 2 | 151 | | 0.87 | 1.72 | 0.23 | 0.99 | |
| WP_003083211.1 | PAAR_G | 86 | 80 | 1 | 80 | | 0.76 | 1.66 | 0.21 | 0.85 | |
| WP_003018233.1 | PAAR_H1 | 173 | 117 | 53 | 169 | | 0.75 | 2.36 | 0.09 | 0.95 | |
| WP_012425654.1 | PAAR_H2 | 121 | 119 | 2 | 120 | | 0.73 | 2.65 | 0.12 | 0.95 | |
| WP_052482490.1 | PAAR_H3 | 127 | 106 | 17 | 122 | | 0.61 | 3.28 | 0.18 | 0.94 | |
