# Supplementary figures and images for: PAAR Proteins Are Versatile Clips That Enrich the Antimicrobial Weapon Arsenals of Prokaryotes
Source: mSystems. 2021 Dec 7;6(6):e00953-21. doi: 10.1128/mSystems.00953-21 (PMC8651086; doi:10.1128/mSystems.00953-21)

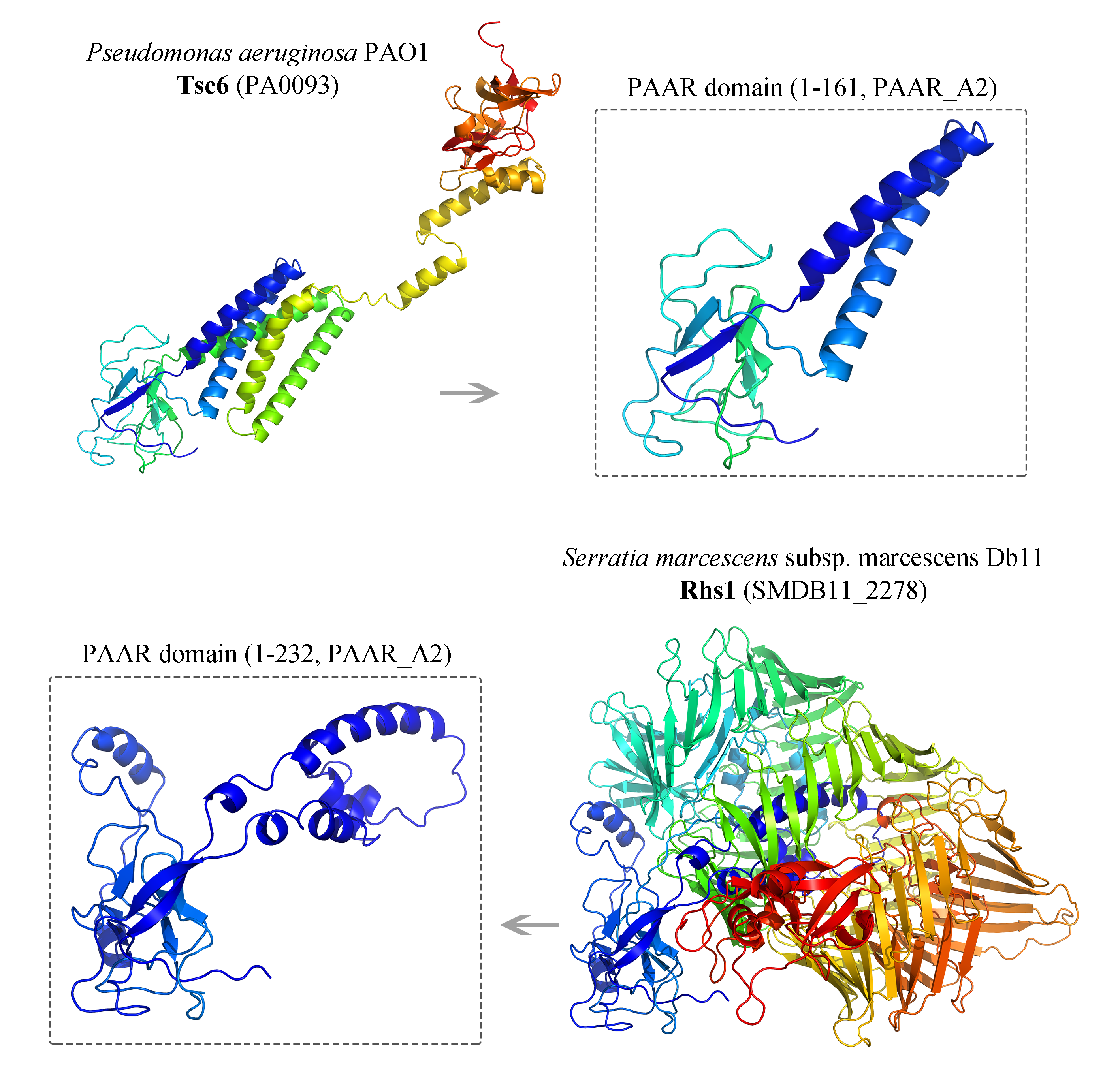

Supplement: FIG S1 [file msystems.00953-21-sf001.tif]

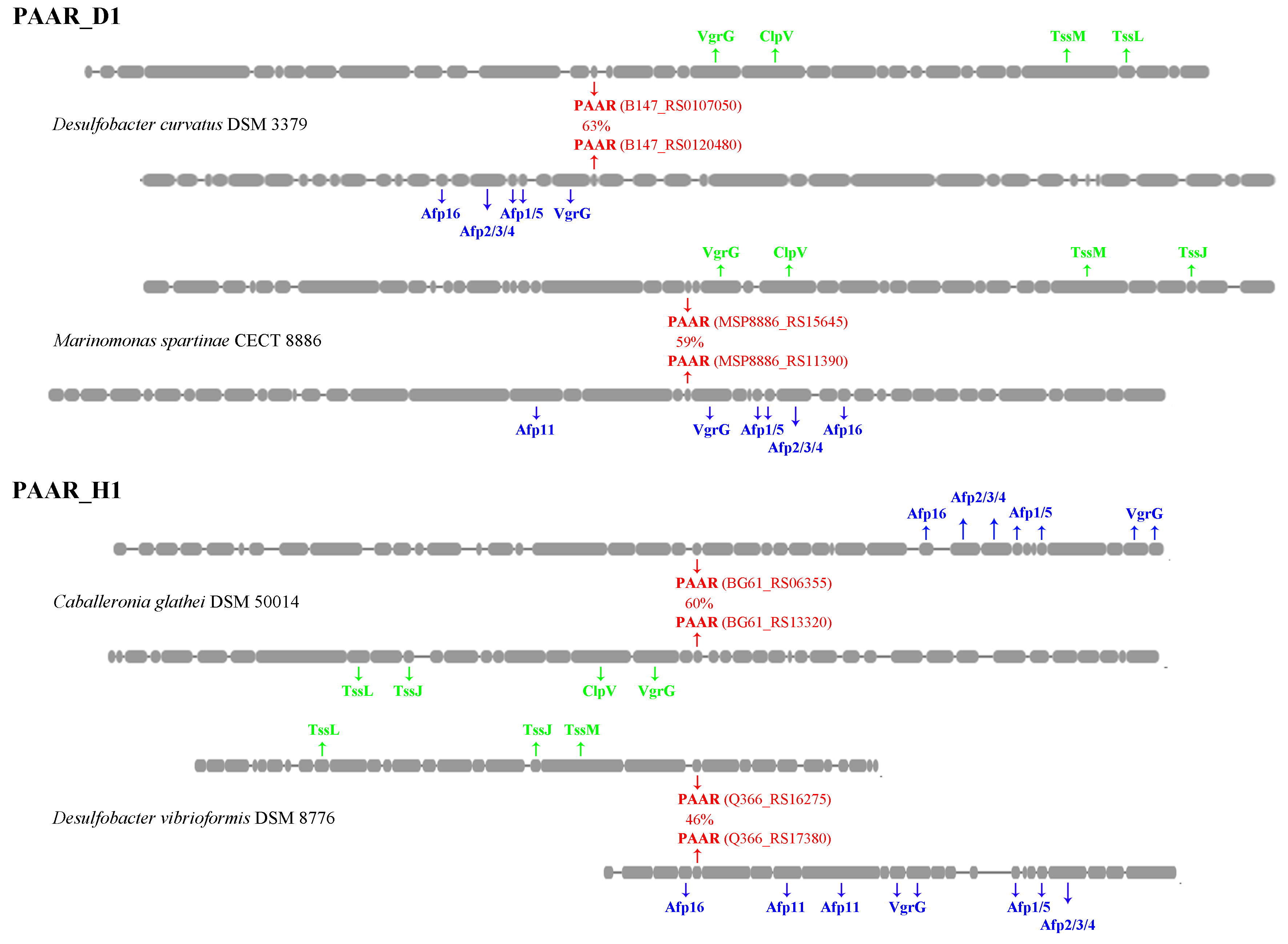

Supplement: FIG S2 [file msystems.00953-21-sf002.tif]

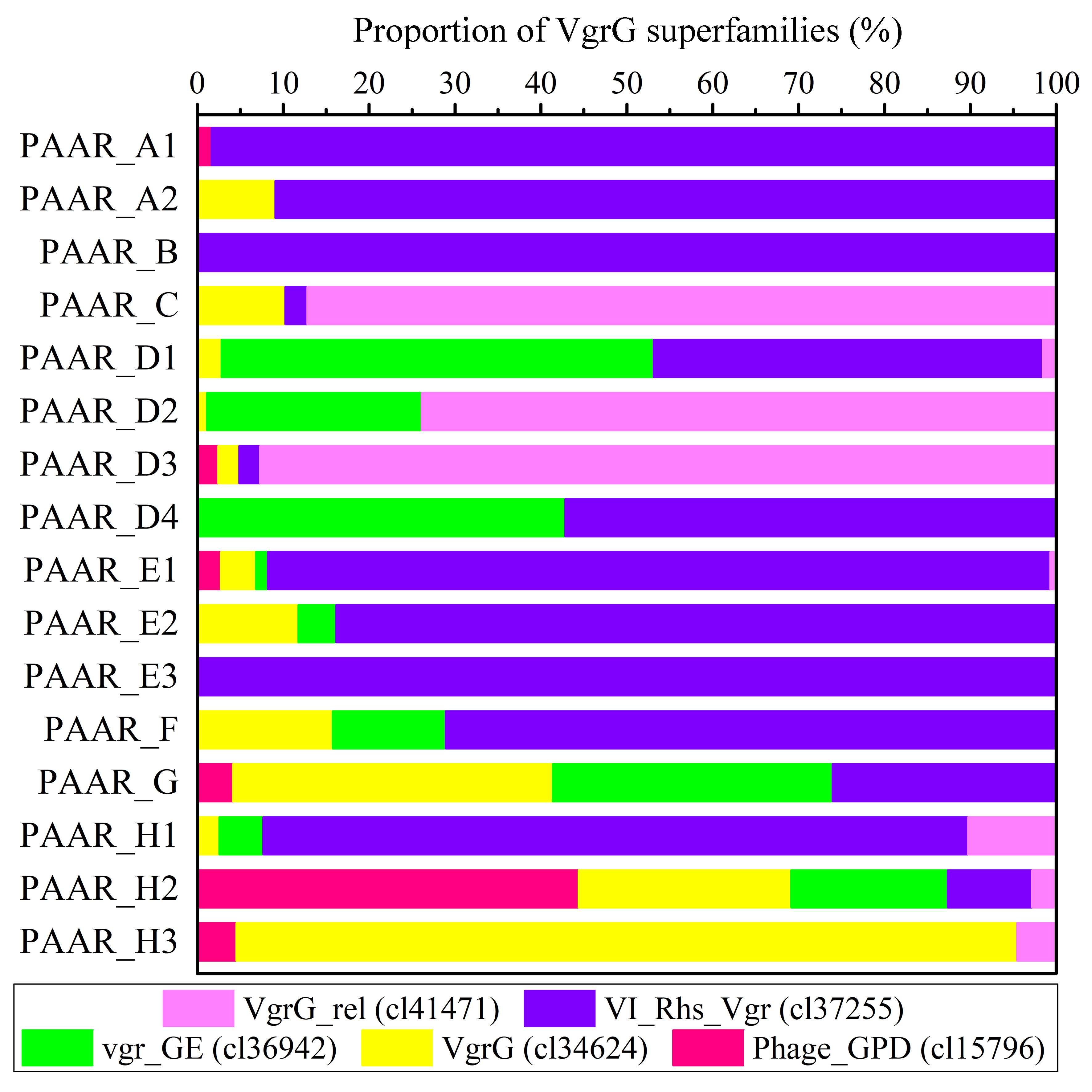

Supplement: FIG S3 [file msystems.00953-21-sf003.tif]

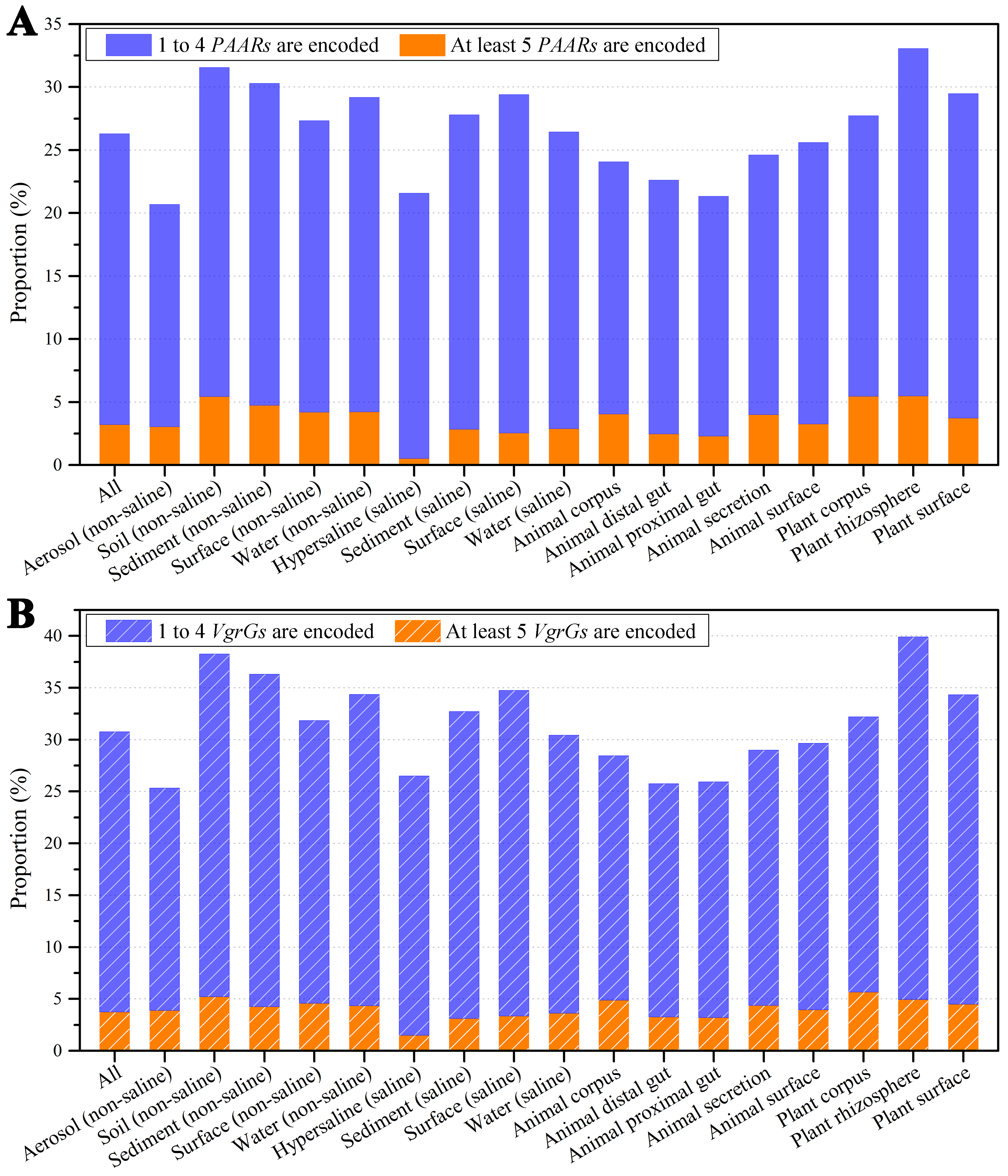

Supplement: FIG S4 [file msystems.00953-21-sf004.tif]

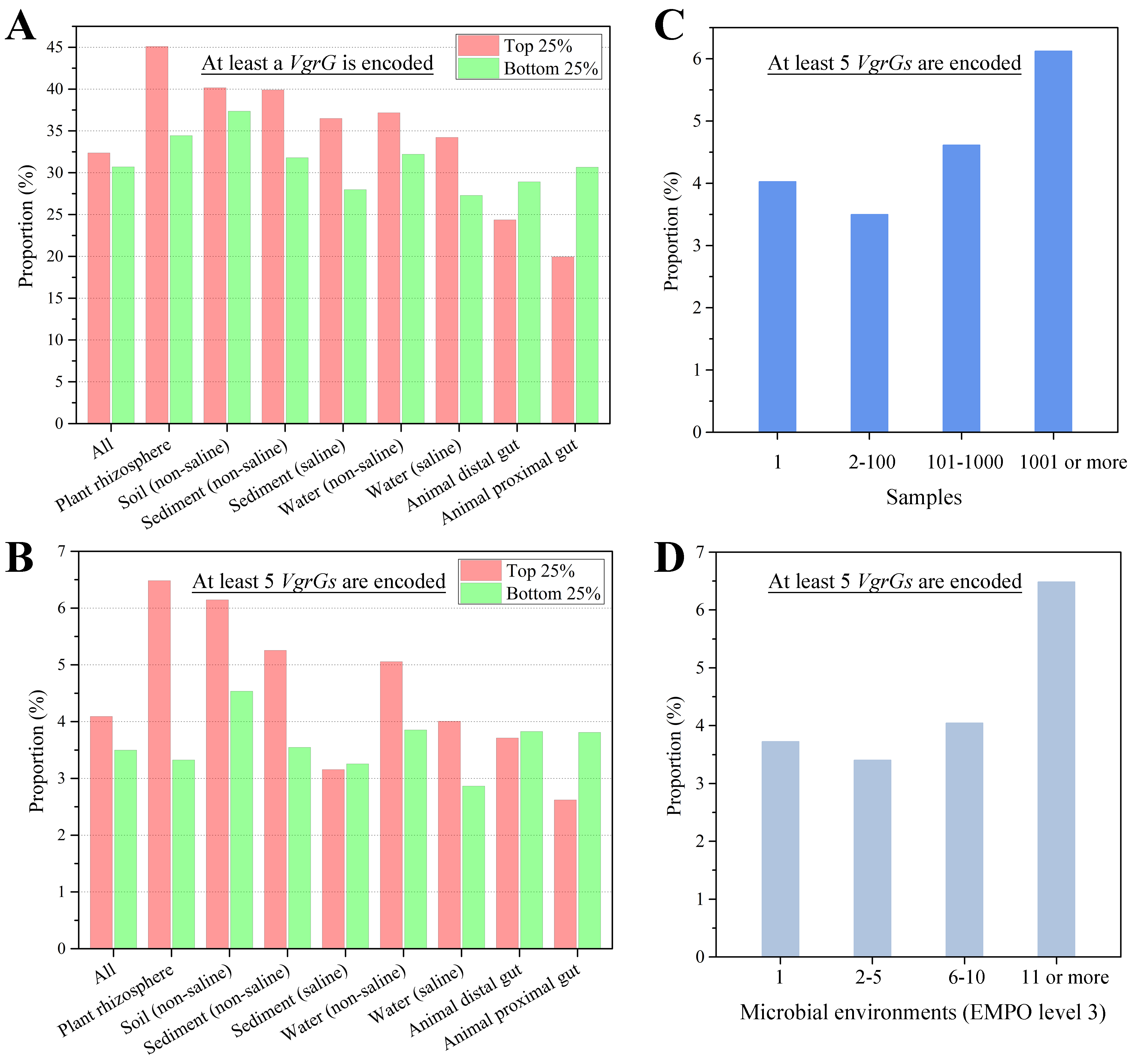

Supplement: FIG S5 [file msystems.00953-21-sf005.tif]
